# Supplementary material for: Surgical treatment in the chronic phase for uncomplicated Stanford type B aortic dissection
Source: PLoS One. 2024 Feb 23;19(2):e0298644. doi: 10.1371/journal.pone.0298644 (PMC10890721; doi:10.1371/journal.pone.0298644)
Supplement: S2 Data — (DOCX) [file pone.0298644.s003.docx]

Study protocol

The treatment way and indication or timing for late aortic events in Stanford type B aortic dissection

Research Institution and Principal Investigator:

Akihito Matsushita

Chief medical director

Department of cardiovascular surgery, Seikeikai Chiba Medical Center

1-7-1, minami-cho, chuo-ku, chiba-shi

Tel: 043-261-5111

FAX：043-261-2305

E-mail:afwa7417@chiba-u.jp

Scheduled clinical research period：After ethics review approval in 2019 to April 2023

Date of preparation：May 11, 2019 Draft plan 1^st^ version prepared

June 20, 2019 Seikeikai Chiba Medical Center Ethics Review committee approval

April 20, 2023 Second version of draft plan prepared

May 12, 2023 Seikeikai Chiba Medical Center Ethics Review Committee approval

Glossary

**Aortic disscetion**: In the aorta, which has a three-layered structure, blood flows into the middle layer of the aorta(the tunica media) for some reason, causing the layers to separate (dissection).

**Stanford classification**: Aortic dissection was classified as type A if dissection involve the ascending aorta. Aortic dissection was classified as type B if dissection did not involve the ascending aorta.

**Phase of disease**: The acute phase is within 2 weeks after the onset of the disease, the chronic phase is after 3 months and the subacute phase is in between.

**Entry, true lumen, false lumen**：Entry in dissection refers to the communication path between the original vascular lumen (true lumen) and the newly created intramedial vascular lumen (false lumen) caused by a tear in intima. The entry closest to the center is called the first entry.

**Malperfusion** : Various disorders caused bya insufficient blood flow due to disorders such as stenosis, occlusion, and rupture of branch vessels from large vessels due to organ reflux disorders and dissection. Ischemia at any site has a high fatality rate.

**Endovascular repair (with Stent-graft) ：**A treatment method in which a graft (artificial blood vessel) supported by a metal flamework (stent) is placed in the area of the aneurysm without surgically incising the surrounding tissue.

＜Table of contents＞

1. Background　　　　　　　　　　　　　　　　　　　　　　　　　　　　　　　P4
2. Purpose 　　　　　　　　　　　　　　　　　　　　　　　　　　P4
3. Study Subjects 　　　　　　　　　　　　　　　　　　　　　　　　　　　P4
4. Methods 　　　　　　　　　　　　　　　　P5-6
5. Study period　　　　　　　　　　　　　　 　　　　　　　　　　　　　　　　P6
6. Risk/benefit assessment 　　　　　　　　　　　　　　　　　　　　　　　　　P6
7. Procedures for obtaining informed consent 　　　　　　　　　　　　　　　　P6
8. Handling of research in situations of immediate and apparent danger to subjects P6
9. How to deal with health hazards　　　　　　　　　　　　　　　　　　　　 　P7
10. How to protect personal information　　　　　　　　　　　　　　　　　　 　P7
11. Research funding and conflicts of interest　　　 　　　　　　　　　　　　　　P7
12. Possibility of secondary use of materials and information and provision to other research institutions.　　　　　　　　　 　P7
13. Research organization　　　　　　　　　　　　　　　　　　　　　 　　 P7-8
14. Storage of records　　　　　　　 　　　　　　　　　　　　　　　　　　　　　P8
15. How to present research results P8
16. Financial burden or remuneration of research subjects P8
17. Reference materials P8-9

1.　Background

Conservative treatment is the first choice for acute treatment of acute Stanford type B aortic dissection unless rupture and malperfusion are involved. However there are many cases in which the dissection site expand in the chronic phase and requires surgical treatment［1］. Some cases require extensive, highly invasive surgery, several fractional surgeries, and some have sudden death due to aortic rupture. In recent years, stent-graft treatment has been induced, and it has been reported to improve prognosis with preemptive therapy［2］.The guidelines have also been revised to allow consideration of endovascular repair for chronic Stanford type B aortic dissection［3］.There is also a report that the final prognosis improvement effect is poor even if treatment is performed according to the current surgical indication criteria so far［4］. Surgical methods or timing or criteria remain controversial in patients with uncomplicated type B aortic dissection at the chronic phase.

2. Purpose

If the risk factors requiring surgery in the chronic phase of Stanford type B aortic dissection can be identified in the acute phase, it will be possible to improve the remote prognosis through intensive observation and early medical intervention. We have identified risk factors［5］. And we developed a risk score that requires invasive vascular treatment in the chronic phase by combining these factors［6］. As a result, it may be possible to avoid extended surgery and remote death with minimal invasiveness and low risk by performing endovascular repair in the group with risk factors earlier than the current criteria for surgery with thoracotomy. I would like to aim for clinical application of this risk score. By examining the course of treatment in our hospital in detail, we would like to examine whether the treatment timing or criteria and treatment can be tolerated as preventive surgery.

3. Study subjects

1. Participant inclusion criteria：Patients underwent medical treatment for Stanford type B acute aortic dissection in the acute phase at Chiba Central Medical Center or Seikeikai Chiba Medical Center.
2. Participant exclusion criteria：Cases in which surgical treatment was selected due to rupture or malperfusion in the acute phase. Cases with a history of dissection.

4. Methods

Specific research method: Perform statistical analysis using existing data.

Sources of existing data and how they are used

・Patients diagnosed type B aortic dissection who underwent acute treatment have undergone regular outpatient CT examinations even after discharge. In principle, CT examinations are performed 3 months, 6months, and 1 year after discharge, and thereafter CT examinations are performed every other year.

・Evaluate outcomes based on CT examination results and chart surveys up to 2022 and perform statistical analysis.

Role of each facility

Remote surveys have been conducted at chiba central medical center and chiba medical center, and data was obtained from the charts. Statistical analysis at Keio University.

Study design：retrospective observational cohort study

Subject registration：Inform and register the details of the research for cases diagnosed and treated for type B aortic dissection at Chiba central medical center and Seikeikai chiba medical center.

Sample size: The sample size target is set at approximately 100 to 150 cases from previous studies［2, 4, 7］.

Report items and schedule

Case’s background：Gender, age, medical history, underlying disease (hypertension, hyperlipidemia, diabetes, dialysis, chronic respiratory disease, arteriosclerosis obliterans, smoking history, coronary artery disease, thoracic surgery history)

Outpatient blood pressure control status（achievement of antihypertensive treatment targeting systolic blood pressure of 120 mmHg）

Examination：CT image at onset（maximum aortic diameter、false lumen diameter、true lumen diameter, false lumen thrombosis status）

Remote CT images：3 months, 6 months, 12 months after onset and final CT with the same items as above.

Treatment timing and treatment way：Indication of surgery（aortic diameter ≧55㎜, rapid aortic enlargement；5㎜/3months to 10㎜/1eyar, and saccular aneurysmal change）, treatment way, time from dissection onset to aortic events, Details of treatment（operating time, devices, special notes during surgery）, treatment results（hospitalization period, length of stay in ICU, complication, death, cause of death）

We will conduct a data survey of the above items through chart searches from September 2019 to June 2022.

Analysis overview

・Definition of primary endpoint: all-cause mortality

・Definition of secondary endpoint: Aortic-related mortality (death due to rupture, death after dissection-related reoperation, clinical suspicion of aortic death)

・Analysis method

Data will be collected through chart searches, letters, and phone calls. Monitoring will also be requested.

・Statistical analysis method

For event occurrence data, Kaplan-Meier method, log-rank test, and Cox regression analysis were applied. For dichotomous data, the effect of confounding factors on the primary endpoint was corrected by logistic regression analysis and treatment methods and treatment indication criteria were adjusted. Explore the impact on primary and secondary endpoints. In addition, we will compare the evaluation items from the onset of dissection and the indication for surgery according to the treatment methods.

5. Study period

1. Subject enrollment period：October 2004 ～August 2021
2. Subject observation period：October 2004 ～June 2022
3. Research period：After ethics review approval in 2019 ～April 2023

* An annual report will be submitted annually to evaluate deviations from the plan during the research period.

6. Risk/benefit assessment

Benefit：There is no direct benefit to subjects from this study.

Risk： Since this is a retrospective observational study using medical chart data, no new samples will be collected, so there will be no disadvantages.

7. Procedures for obtaining informed consent

During the hospitalization, the subjects were notified of the possibility of secondary use for research purposes of test data obtained during outpatient treatment. The content of this research will also be disseminated in a posted document approved by the ethics committee.

8.　 Handling of research in situations of immediate and apparent danger to subjects

This is a retrospective study using medical chart data, and it cannot be a situation in which the research subjects are in an urgent and obvious crisis.

9.　 How to deal with health hazards

Since this research will study information obtained within the scope of normal medical care (or pathological tissue collected by surgery or biopsy), there will be no invasive behavior for direct research purposes on subjects. Therefore, no health hazards resulting from this research will occur.

10.　How to protect personal information

1）Whether samples can be anonymized and specific individuals can be identified? We anonymize the data. It is not possible to identify a specific individual.

2）Scope of data handling including personal information.

Only the research director. Share data that does not contain personal information with the statistician.

3）Handling of data after withdrawal of consent.

Data after withdrawal of consent will be destroyed.

4）How to manage the correspondence table?

The correspondence table will be stored as data at the Chiba medical center.

11.　 Research funding and conflicts of interest

　This study does not need research funds to view medical record data and perform data analysis. The all investigators have no conflict of interest.

12.　Possibility of secondary use of materials and information and provision to other research institutions.　Not at this time.

13.　 Research organization

| Department of cardiovascular surgery, Chiba Central Medical Center,  Department of cardiovascular surgery, Seikeikai Chiba Medical Center | | |
| --- | --- | --- |
| Senior manager | Wahei Mihara | Inform subjects and obtain informed consent. Treatment. Outpatient observation. |
| Manager | Takashi Hattori | Inform subjects and obtain informed consent. Treatment. Outpatient observation. |
| Chief medical director | Akihito Matsushita | Data acquisition, CT image analysis and statistical processing, Research director. |
| Department of cardiovascular surgery, Juntendo University | | |
| Senior manager | Minoru Tabata | Guidance on statistical analysis, consideration of research results. |
| Department of Preventive Medicine and Public Health, Keio University | | |
| Associate Professor | Yasunori Sato | Guidance on statistical analysis, consideration of research results. |

14.　 Storage of records　　Data is stored only in a locked desktop PC HD in the medical office, which is located in a controlled area within the Seikeikai Chiba Medical Center that is locked from the outside. The storage period shall be 10 years. After the end of the retention period, the data will be promptly deleted.

15.　How to present research results

　It will be presented at an academic conference and submitted to an academic journal during 2019 -2024.

16.　Financial burden or remuneration of research subjects

　No financial burden, no reward.

17.　Reference materials

［1］G.C. Hughes, N.D. Anderson, and R.L. McCann. Management of Acute Type B Aortic Dissection. Journal of Thoracic and Cardiovascular Surgery 2013; 145: S202-7

［2］C.A. Nienaber, S Kische, H Rousseau, et al.　Endovascular Repair of Type B Aortic Dissection Long-term Results of the Randomized Investigation of Stent Grafts in Aortic Dissection Trial. Circulation Cardiovascular Interventions 2013; 6: 407-416

［3］R Erbel, V Aboyans, C Boileau, et al. 2014 ESC Guidelines on the diagnosis and treatment of aortic diseases. European Heart Journal 2014; 35: 2873-2926

［4］X Lou, EP Chen, YM Duwayri, et al. The Impact of Thoracic Endovascular Aortic Repair on Long-Term Survival in Type B Aortic Dissection. Annals Thoracic Surgery 2018; 105: 31-39.

［5］A Matsushita, T Hattori, Y Tsunoda, et al. Impact of initial aortic diameter and false-lumen area ratio on Type B aortic dissection prognosis. Interact Cardiovasc Thorac Surg. 2018; 26: 176-182.

［6］A Matsushita, M Tabata, W Mihara, et al. Risk Prediction Score System for Late Aortic Events in Patients with Uncomplicated Stanford Type B Aortic Dissection. Journal of Thoracic and Cardiovascular Surgery. 2019; impress

[7] Boufi M, Patterson BO, Loundou AD, et al. Endovascular versus open repair for chronic type B aortic dissection treatment: a meta-analysis. Ann Thorac Surg 2019; 107:1559-70.
