# Supplementary material for: Surgical treatment in the chronic phase for uncomplicated Stanford type B aortic dissection
Source: PLoS One. 2024 Feb 23;19(2):e0298644. doi: 10.1371/journal.pone.0298644 (PMC10890721; doi:10.1371/journal.pone.0298644)
Supplement: S3 Data — (DOCX) [file pone.0298644.s004.docx]

研究計画書

Stanford B型急性大動脈解離における遠隔期血管手術適応症例における

加療時期と加療法についての研究

研究責任者：誠馨会千葉メディカルセンター　心臓血管外科　主任医長

　　　　　　松下明仁

　　　　　　住所：千葉市中央区南町1-7-1

　　　　　　電話番号：043-261-5111（内線：8173）

　　　　　　FAX番号：043-261-2305

E-mail:afwa7417@chiba-u.jp

緊急連絡先：松下明仁　090-5298-2354

臨床研究実施予定期間：2019年倫理審査承認後　から　2023年4月

作成日： 2019年 5月11日　計画書案　第1版作成

　　　　 2019年 6月20日　誠馨会千葉メディカルセンター倫理審査委員会承認

　　　　 2023年4月20日　計画書案　第2版作成

　　　　 2023年5月12日　誠馨会千葉メディカルセンター倫理審査委員会承認

用語・略語

**大動脈解離**: 3層構造を作っている[大動脈](https://ja.wikipedia.org/wiki/%E5%A4%A7%E5%8B%95%E8%84%88)のうち、何らかのきっかけで真ん中の層の膜（中膜）に[血流](https://ja.wikipedia.org/wiki/%E8%A1%80%E6%B5%81)が入り込み、層構造が別々に剥がれていく（解離）疾患。

**Stanford分類**: 上行大動脈に解離が及んでいるA型と及んでいないB型に分類している。

**病期**:疾患発症後2週間以内を急性期、3ヶ月以降を慢性期、その中間を亜急性期としている。

**Entry、真腔、偽腔**：解離におけるEntryとは、内膜に裂孔を生じ、本来の血管腔（真腔）と新しくできた中膜内血管腔（偽腔）の交通路のこと。最も中枢側のEntryをfirst entryと呼ぶ。

**Malperfusion**:臓器還流障害、解離により大血管からの分枝血管が狭窄、閉塞、断裂などの障害を起こし血流が不足することで生じる種々の障害。どの部位の虚血であっても致死率が高い。

**ステントグラフト加療：**金属製の骨組み(ステント)に支えられえたグラフト(人工血管)を周辺組織を外科的に切開することなく、動脈瘤の範囲に留置する加療方法。

＜目次＞

1. 研究の背景　　　　　　　　　　　　　　　　　　　　　　　　　　　　　　　P4
2. 研究の目的および意義　　　　　　　　　　　　　　　　　　　　　　　　　　P4
3. 研究対象者の選定　　　　　　　　　　　　　　　　　　　　　　　　　　　　P4
4. 研究の方法および研究の科学的合理性の根拠　　　　　　　　　　　　　　　　P4-6
5. 研究実施期間　　　　　　　　　　　　　　　　　　　　　　　　　　　　　　P6
6. 予測される利益と不利益　　　　　　　　　　　　　　　　　　　　　　　　　P6
7. インフォームド・コンセントを受ける手続き　　　　　　　　　　　　　　　　P6
8. 研究対象者に緊急かつ明白な危機が生じている状況における研究の取り扱い　　P6
9. 健康被害発生時の対処方法　　　　　　　　　　　　　　　　　　　　　　　　P6
10. 個人情報の保護方法　　　　　　　　　　　　　　　　　　　　　　　　　　　P6-7
11. 研究資金および利益相反　　　　　　　　　　　　　　　　　　　　　　　　　P7
12. 資料・情報の2次利用および他研究機関への提供の可能性　　　　　　　　　　P7
13. 研究組織　　　　　　　　　　　　　　　　　　　　　　　　　　　　　　　　P7
14. 記録等の保管　　　　　　　　　　　　　　　　　　　　　　　　　　　　　　P7
15. 研究成果の発表方法 P7
16. 研究対象者の経済的負担又は謝礼 P7
17. 参考資料・文献リスト P7-8

1.　研究の背景

Stanford B型急性大動脈解離の急性期加療は破裂、Malperfusion（臓器還流障害）を伴っていなければ保存的加療が第一選択である。（通常2~3週間の入院で血圧調節、疼痛コントロールを行った後に退院としている。）ただし、慢性期に解離部位が拡大をきたし手術加療を必要とする症例も多く認めている［1］。また、広範囲動脈拡大のために侵襲の大きい拡大手術や数回に分けた分期手術を必要とする症例もあり、大動脈破裂や破裂が疑われる突然死を起こす症例もある。急性期を良好に経過した症例でも予後不良となりうる疾患である。近年、ステントグラフト加療が導入され、解離発症後1年以内の加療によりEntryを閉鎖することで血管のリモデリングを促し、予後を改善するという報告もあり［2］、ガイドライン上も慢性期Stanford B型解離に対するステントグラフト加療は考慮の余地ありと変更されてきている［3］。ステントグラフト加療はこれまでの開胸手術に比較して低侵襲、低リスクと考えられている。これまでの手術適応基準で加療を行っても最終的な予後改善効果が乏しいという報告もあり［4］、加療時期、加療法については議論がある。

2. 研究の目的および意義

Stanford B型大動脈解離は慢性期にも密な外来通院と定期的なCT検査で手術時期を逸しないように注意して観察する必要があるが、現実的にはあまりに密な検査は困難である。慢性期に手術を必要とする危険因子を急性期に同定できれば特に重点的な観察や早期の加療介入で遠隔予後の改善が期待される。これまでに危険因子を同定し［5］、その因子の組み合わせで慢性期に血管に侵襲的な加療を要するリスクスコアを開発した［6］。これにより開胸での手術適応基準よりも早期に危険因子保持群にステントグラフト加療を行うことで低侵襲、低リスクで拡大手術や遠隔死亡を回避できる可能性がある。このリスクスコアの臨床での応用を目指したい。ただし、これまでの加療適応基準を下げるという側面を有する予防的な加療を行うには、より低侵襲であることが求められる。当院でのこれまでの加療経過を詳細に検討することで、その加療時期、加療法について予防的手術として許容できるものかを検討したい。

3. 研究対象者の選定

1. 対象選択基準：千葉中央メディカルセンター、千葉メディカルセンターでStanford B型解離の急性期保存的加療を行った症例。
2. 対象除外基準：急性期に破裂やmalperfusionにより手術加療を選択した症例は除く。解離の既往のある症例は除く。

4. 研究の方法および研究の科学的合理性の根拠

具体的研究方法：既存データを使用して統計解析を行う。

既存データの出典と利用方法

・これまでにStanford B型大動脈解離の診断で急性期加療を行った患者は退院後も定期的な外来通院でCT検査を施行している。原則的には退院後3ヶ月、半年、1年でCT検査を撮影し、その後は1年おきにCT検査を撮影している。

・2022年までのCT検査結果とカルテ調査でアウトカムを評価し統計解析を行う。

各施設の役割

千葉中央メディカルセンター、千葉メディカルセンターでこれまでも遠隔調査を施行しており、そのカルテからデータを取得。慶應義塾大学で統計解析を行う。

観察研究デザイン：データ取得の向き（後ろ向き）　研究の種類（コホート研究）

サンプルサイズとして過去の類似研究から100 ~ 150症例を目標とした。［2，4，7］

具体的方法

被験者登録：千葉中央メディカルセンター、千葉メディカルセンターでB型解離を診断し加療を行った症例に対して研究内容を周知して登録する。

観察・検査・調査・報告項目とスケジュール

患者背景：性別、年齢、既往、基礎疾患（高血圧、高脂血症、糖尿病、透析、慢性呼吸疾患、閉塞性動脈硬化症、喫煙歴、冠動脈病変、胸部手術歴）

外来での血圧コントロール状況（収縮期血圧120mmHgを目標とした降圧加療の達成）

検査：発症時CT画像（発症時大動脈最大径、偽腔径、真腔径、偽腔血栓状態）

　　　遠隔CT画像：発症後3ヶ月、6ヶ月後、12ヶ月後と最終CTで上記と同じ項目

加療時期と加療法：解離関連手術適応（大動脈最大径≧55㎜、急激な血管径の変化；5㎜/3か月もしくは10㎜/1年、嚢状瘤形成）に対する加療方法、加療適応までの期間、加療適応となってから手術までの期間、加療内容（手術時間、使用デバイス、手術時特記事項）、加療結果（加療時入院期間、ICU滞在日数、合併症、死亡、死亡原因）

2019年9月～2022年6月に上記項目をカルテ検索によりデータ調査する。

解析の概要

・主要評価項目の定義

主要評価項目として全死亡

・副次評価項目の定義

副次評価項目として血管関連死亡（破裂による死亡）、解離関連再手術、解離関連再手術適応があるが手術を拒否している状態。

・解析方法

データ収集はカルテ検索、手紙・電話連絡で行う。モニタリングも施行を依頼する。

・統計解析方法

イベント発生データは、Kaplan-Meier法, log-rank検定、Cox回帰分析を適用し、２値データに関しては、ロジスティック回帰分析により主要評価項目に対する交絡因子の影響を修正し、加療方法と加療時期が主要評価項目、副次評価項目に与える影響を探索する。また加療方法別に解離発症からと手術適応からの評価項目を比較する。

5. 研究実施期間

1. 被験者登録期間：2004年10月～2021年8月
2. 被験者観察期間：2004年10月～2022年6月
3. 研究実施期間：2019年倫理審査承認後　から　2023年4月

* 研究継続中は毎年、計画からの逸脱がないかを確認するために年次報告を提出する。

6. 予測される利益と不利益

利益：本研究により被験者が直接受けることができる利益はない。

不利益： カルテデータを用いた後ろ向き観察研究であり、新たな試料は採取しないため不利益は生じない。

7. インフォームド・コンセントを受ける手続き

被験者には入院中、外来で加療に際して行った検査データについて研究目的に2次利用する可能性を掲示で伝えている。今回の研究内容についても同様に倫理委員会で承認の得られた掲示文書で周知する。

8.　研究対象者に緊急かつ明白な危機が生じている状況における研究の取り扱い

カルテデータを用いた後ろ向き研究であり、研究対象者に緊急かつ明白な危機が生じている状況にはなりえない。

9.　健康被害発生時の対処方法

本研究は、通常の診療の範囲内で得られた情報（あるいは、手術又は生検によって採取した病理組織）を研究対象とするので、被験者に対する直接的な研究目的の侵襲性を伴う行為は一切行わない。そのため、本研究に起因する健康被害が発生することはない。

10.　個人情報の保護方法

1）試料等の匿名化および特定の個人を識別できるか否か。データは匿名化してあつかう。特定の個人は識別できない。

2）個人情報を含むデータの取扱者の範囲　研究責任者のみ。個人情報を含まないデータを統計責任者と共有する。

3）同意撤回後のデータの取り扱いについて　同意撤回後のデータは研究上破棄する。

4）対応表の管理方法　対応表は千葉メディカルセンターでデータ保管する。

11.　研究資金および利益相反

　当研究ではカルテデータを閲覧しデータ解析を行うために、研究資金の必要性はない。当研究に関わる研究者の利益相反状況は別に示すが、研究責任者に利益相反はない。

12.　資料・情報の2次利用および他研究機関への提供の可能性

　　現時点ではない。

13.　研究組織

| 千葉中央メディカルセンター、千葉メディカルセンター　心臓血管外科 | | |
| --- | --- | --- |
| 主任部長 | 三原　和平 | 被験者への説明と同意の取得。被験者の加療、外来観察。 |
| 部長 | 服部　隆司 | 被験者への説明と同意の取得。被験者の加療、外来観察。 |
| 主任医長 | 松下　明仁 | データ取得、CT画像解析と統計処理。研究責任者。 |
|  |  |  |
| 順天堂大学医学部付属順天堂医院　心臓血管外科 | | |
| 部長 | 田端　実 | 統計解析指導、研究結果考察判断 |
|  |  |  |
| 慶應義塾大学医学部衛生学公衆衛生学 | | |
| 准教授 | 佐藤　泰憲 | 統計解析指導、研究結果考察判断 |

14.　記録等の保管　データを千葉メディカルセンター内の外部からは施錠されている管理区域内にある医局内の暗号ロックされたデスクトップパソコンHD内にのみ保管する。保管期間は10年間とする。保管期間終了後にはデータは速やかに消去する。

15.　研究成果の発表方法

　2019年~2024年中に学会発表、学会誌に提出する。

16.　研究対象者の経済的負担又は謝礼

　経済的負担はない、謝礼もない。

17.　参考資料・文献リスト

［1］G.C. Hughes, N.D. Anderson, and R.L. McCann. Management of Acute Type B Aortic Dissection. Journal of Thoracic and Cardiovascular Surgery 2013; 145: S202-7

［2］C.A. Nienaber, S Kische, H Rousseau, et al.　Endovascular Repair of Type B Aortic Dissection Long-term Results of the Randomized Investigation of Stent Grafts in Aortic Dissection Trial. Circulation Cardiovascular Interventions 2013; 6: 407-416

［3］R Erbel, V Aboyans, C Boileau, et al. 2014 ESC Guidelines on the diagnosis and treatment of aortic diseases. European Heart Journal 2014; 35: 2873-2926

［4］X Lou, EP Chen, YM Duwayri, et al. The Impact of Thoracic Endovascular Aortic Repair on Long-Term Survival in Type B Aortic Dissection. Annals Thoracic Surgery 2018; 105: 31-39.

［5］A Matsushita, T Hattori, Y Tsunoda, et al. Impact of initial aortic diameter and false-lumen area ratio on Type B aortic dissection prognosis. Interact Cardiovasc Thorac Surg. 2018; 26: 176-182.

［6］A Matsushita, M Tabata, W Mihara, et al. Risk Prediction Score System for Late Aortic Events in Patients with Uncomplicated Stanford Type B Aortic Dissection. Journal of Thoracic and Cardiovascular Surgery. 2019; impress

［7］Boufi M, Patterson BO, Loundou AD, et al. Endovascular versus open repair for chronic type B aortic dissection treatment: a meta-analysis. Ann Thorac Surg 2019; 107:1559-70.
