# Supplementary material for: Surgical treatment in the chronic phase for uncomplicated Stanford type B aortic dissection
Source: PLoS One. 2024 Feb 23;19(2):e0298644. doi: 10.1371/journal.pone.0298644 (PMC10890721; doi:10.1371/journal.pone.0298644)
Supplement: Attachment — Submitted filename: Response to Reviewers 0803.docx [file pone.0298644.s006.docx]

Reviewer #1: The authors reported their experience with patients affected by uncomplicated chronic Stanford type B aortic dissection, comparing the treatment group (surgical + endovascular) vs. the optimal medical therapy group. After their analysis, they concluded that uncomplicated type B aortic dissection should be treated, choosing the best option according the patient’s clinical status. I think that their work does not bring nothing new and interesting in the current literature. Better designed papers have been recently published (PMID: 36646334; PMID: 36603666; PMID: 36334259; PMID: 35944731). Moreover, the manuscript could benefit of an extensive English revision. It is hard to follow.

⇒We appreciate your comment.　We checked recently published papers (PMID: 36646334; PMID: 36603666; PMID: 36334259; PMID: 35944731).　These studies were well design and of excellent quality. Two of them (PMID:36603666; PMID:35944731) focus specifically on the timing of TEVAR and provide information that TEVAR in the hyperacute phase should be avoided. A two-center retrospective review (PMID: 36646334) revealed TEVAR at the initial admission had lower rates of unplanned readmission and need for unplanned surgery rather than only medication for uncomplicated type B aortic dissection. In contrast, the large cohort study (PMID: 36334259) revealed initial TEVAR was not associated with improved mortality or reduced hospitalizations or aortic interventions over a period of 5 years. In common with all these papers, they stated that there is still a shortage of research in this area and that more studies with more significant numbers of cases, more extended study periods, and studies to contrast are needed. Although our study differs from these studies in subject matter and perspective, and as the points out, does not represent a groundbreaking novelty, we hope that our experience in treating more than 100 consecutive cases over 17 years at two institutions will have some impact on this field.

After revising the paper as instructed by the reviewers, we re-performed the English editing. We appreciate again for the reviewer’s remarks.

I suggest few comments to improve the manuscript.
- The material and methods paragraph should not include results! (Remove citation for Figure 2 and Figure 3).

⇒We appreciate your comment. We removed citations for Figures and Tables from the material and methods paragraph.

- In the treatment group, the surgical and endovascular strategies varied during the years. It could be better to compare the outcomes considering the two time periods (line 137) cited in the manuscript.

⇒We appreciate your comment. We compared the outcomes for all patients and each treatment method group depending on periods; then a new Figure 4 was created. As a result, there was no improvement in treatment outcomes by period (Line 309-315, Figure 4).

- Avoid ST as abbreviation. There are too many in the text. Moreover, the authors include in the ST group also the TEVAR patients. It would be more appropriate to distinguish this group as Treatment Group.

⇒We appreciate your comment. We avoided the abbreviation ST and changed it to the treatment group.

- How do the authors discuss their results compared with those of Weissler and colleagues (PMID: 36334259)?

⇒We appreciate your comment. Weissler and colleagues reported initial TEVAR was not associated with improved mortality or reduced hospitalizations or aortic interventions over 5 years as the main results. They also reported in a sensitivity analysis that included deaths within the first 30 days, initial TEVAR was associated with low mortality. Included in this sensitivity study were 752 cases(9.54%) who died within 30 days of initial hospitalization in UTBAD, which was an exclusion criterion in the study design as all patients could not be TEVAR within 30 days. Naturally, we determined that this sensitivity study was not the main result and should not be included in the discussion. In our experience, patients with complicated TBAD could have an unfortunate outcome in initial hospitalization. We believe that approximately 10% of UTBAD patients do not die initially, and only one death was confirmed in our study. This may be due to selection bias and differences in ethnicity and nationality. However, it is also possible that this is a database study and does not accurately capture the reality of the UTBAD. The PMID 36646334 study also showed only one death during initial UTBAD hospitalization. The main result alone is that initial TEVAR does not differ in outcome, and this point was added as a reference (Line 356-359, 440-443).

- A more detailed comparison of the three group (Surgical, endovascular, and OMT) could be more interesting: inclusion of early complications (neurological adverse events, AKI, rupture after intervention)

⇒We appreciate your comment. We added a stroke case in TEVAR as a neurological adverse event and reported AKI in both groups (Line 161-164, 261-264). Complications, including AKI, seemed more common in open surgery than in TEVAR. However, all patients with AKI were discharged without needing for dialysis (Line 265-266). There were no cases of in-hospital rupture after the intervention. We also reported aortic-related mortality in long-term outcomes. The breakdown of this information was added (Line 274-278).

- The manuscript would be easier and smooth to read if the authors reduce the number of abbreviations.

⇒We appreciate your comment. We reduced the number of abbreviations; from 13 to 10 (Line 63-72).

- The discussion paragraph should be improved.

⇒We appreciate your comment. We have added a discussion of the new paper (PMID: 36334259) as above and added to the discussion the outcomes of the change in treatment strategy (Line 337-340, 356-359).

Reviewer #2: This study compares the mortality rates of patients with chronic uncomplicated type B aortic dissection undergoing surgical (open or TEVAR) vs medical treatment. In order to make the groups comparable, patients without aortic events (i.e. rapid aortic growth, aneurysmatic evolution, saccular aneurysm development) were excluded. So the medical treatment group was composed basically of patients who refused the intervention or were not deemed operable. The authors found that aortic diameter above 55 mm was the main predictor of mortality both in patients undergoing surgical treatment and in the medical treatment group. The methodology is sound and the article is overall well written.

Here are my review suggestions:
line 208: “zone 2 for one patient with subclavian artery (SCA)–SCA bypass”
was this a left carotid-to-subclavian bypass? if so please correct, else specify more clearly what intervention was carried out.
⇒We appreciate your comment. In this case, we performed a right axillary artery –left axillary artery bypass. We revised this sentence(Line 218-219).

209: “zone 2 for three patients with simple Lt. SCA coil embolization” what does Lt stand for? if it is “left” please spell out.

⇒We appreciate your comment. We revised it as you indicated (Line 219).
